# The Application of the Adult Self-Report and the Adult Behavior Checklist Form to Chinese Adults: Syndrome Structure, Inter-Informant Agreement, and Cultural Comparison

**DOI:** 10.3390/ijerph18126352

**Published:** 2021-06-11

**Authors:** Jianghong Liu, Fanghong Dong, Christopher M. Lee, Jenny Reyes, Masha Ivanova

**Affiliations:** 1 Department of Family and Community Health, University of Pennsylvania School of Nursing, Philadelphia, PA 19104, USA; dofa@upenn.edu (F.D.); lcm@sas.upenn.edu (C.M.L.); reyest@wharton.upenn.edu (J.R.); 2 Department of Psychiatry, University of Vermont, Burlington, VT 05405, USA; masha.ivanova@med.uvm.edu

**Keywords:** psychopathology, ASEBA, ASR, ABCL, adult behavior, psychometric, cultural comparison

## Abstract

Given the global public health burden of mental illness, there is a critical need for culturally validated psychopathology assessment tools that perform well in diverse societies. This study examines the psychometric properties of the Adult Self-Report (ASR) and Adult Behavioral Checklist (ABCL) from the Achenbach System of Empirically Based Assessments in adults in China. Chinese adults (N = 1276) and their spouses completed the ASR and ABCL, respectively. We conducted confirmatory factor analysis on 99 ASR items and 93 ABCL items. Estimators of model fit confirmed that both measures demonstrated excellent fit (e.g., root mean square error of approximation = 0.016 and 0.018, respectively). Syndrome loadings on both measures were satisfactory but generally higher on the ASR. Neither gender nor education had significant effects, but there were informant x gender effects on most problem scales. Cross-informant agreement correlations between the ASR and ABCL were medium to large. Findings from this novel sample of Chinese adults are consistent with previous validation studies supporting the dimensionality, syndrome structure, gender differences, and inter-informant agreement of the ASR and ABCL. Our findings contribute to the cross-cultural understanding of mental health assessment and offer a psychometrically sound approach to measuring adult psychopathology in Chinese populations.

## 1. Introduction

The comprehensive measurement of adult psychopathology is an inherently important aspect of preventing or mitigating negative long-term individual and societal outcomes associated with the trajectory of mental illness. Negative outcomes of mental disorders include long-term effects on occupational functioning and reductions in earning potential and the labor force; healthcare and behavioral healthcare resource utilization and spending; and involvement in the criminal justice system [1,2,3]. As such, it is critical to have available validated instruments that can inform the prediction, identification, and management of psychopathology in adulthood.

One such instrument is the Achenbach System of Empirically Based Assessment (ASEBA), one of the most widely used and rigorously validated measures to assess behavioral, emotional, and social problems and adaptive functioning in individuals of all ages [4,5,6,7]. The Adult Self-Report (ASR) and Adult Behavior Checklist (ABCL), components of the ASEBA, are specifically designed to assess psychopathology in individuals aged 18 to 59. Ratings of problem items are summed to yield scores on eight statistically derived narrow-band syndrome scales (e.g., Anxious/Depressed), three broad-band scales (Internalizing, Externalizing, and Total Problems), six DSM-oriented scales, as well as a Friends and Spouse/Partner scale [8]. While the ASR is utilized for self-assessment, the ABCL obtains ratings from an adult closely associated with the subject to provide greater insight into the behavioral problems and psychopathology of the target.

Though the ASR and ABCL have been validated and performed consistently in thousands of adults, cross-cultural differences in phenotypic expressions of psychopathology in these instruments have garnered little attention in the literature. Potential differences in economic, social, and cultural factors as well as the expression of specific psychopathology among disparate cultures/ethnic groups suggest that quantitative assessment methods may not necessarily measure theoretical constructs in the same way in different societies (i.e., lack of measurement invariance) [9]. This hinders our ability to generalize findings about psychopathology constructs, thereby also limiting our full understanding of psychopathological traits and their individual and public health impact. As the ASR/ABCL syndrome structures were primarily derived from U.S. samples, it is necessary to validate the instruments in different societies to ensure its value as a tool to better understand and measure psychopathology in an increasingly diverse global population.

Research into the cross-cultural generalizability of the ASR/ABCL, though limited, has yielded informative findings. Individual society studies include Mahr et al. [10] and Corff et al. [11], conducted in France and Canada, respectively. Mahr et al. [10] performed confirmatory factor analyses (CFAs) to verify the factor structure of the French version of the ASR using self-ratings from university students in France (N = 905), with the analyses yielding the expected ASR 8-syndrome model (RMSEA = 037). Corff et al. [11] evaluated the equivalence of the French-Canadian version of the ASR to the original English version by administering both assessments to bilingual university students in Canada (N = 251), with results supporting the equivalence of the two instruments. Ivanova et al. [12,13] used CFA to test the generalizability of the ASR/ABCL 8-syndrome model in 29 societies (N = 17,152), including Africa, Asia, South America, and Eastern, Northern, and Western Europe. Collateral ratings were tested in 17 of those societies (N = 8582), including a subset of the current Chinese sample. Despite respondents’ differences in language, culture, religion, geographic and geopolitical backgrounds, CFAs of the 8-factor model of traits operationalizing adult psychopathology were remarkably consistent, with root mean square error of approximation (RMSEA) estimates ranging from 0.016 to 0.034, indicating good to excellent fit across all societies.

After excluding those Ivanova samples that were demographically limited, Rescorla et al. [14,15] analyzed ASRs from 18 societies (N = 12,217), ABCLs from 14 societies (N = 8322), and ASR–ABCL cross-informant agreement for 14 societies (N = 8302). Notable similarities were present, but cultural differences were also apparent. Problem scale score means were higher on the ASR than the ABCL in every society, but the size of the differences varied across societies. Items with the most similarity across cultures included those related to worrying, being nervous or tense, lacking self-confidence, having difficulty concentrating, having difficulty with decision making, being argumentative, and irritability [14]. Most societal effect sizes were small and ranged from 2–5% [14]. Individual differences rather than societal differences accounted for most of the variation in problem scores. Cross-informant correlations for problem scale scores averaged 0.47 but varied widely across societies [15].

In order to better understand how ethnic and cultural factors influence the development and expression of psychopathological traits, more research is needed examining the cross-cultural generalizability of commonly used adult psychopathology measures. To that end, we examined Chinese versions of the ASR and ABCL with respect to their syndrome structure, effects of gender, education, and informant on scale scores, and cross-informant agreement. Furthermore, we compared our ASR scores with ASR scores from the 17 societies in the Rescorla et al. [14] international sample, and our ABCL scores with ABCL scores for Rescorla et al.’s [15] 14-societies sample. We hypothesized that the syndrome structure of the ASR and ABCL would be supported in self and collateral ratings of adults in China. We also hypothesized that the ASR/ABCL syndrome structure would be universal across gender and education groups, and we further hypothesized that there would be cultural differences when comparing Chinese ASR and ABCL scores to those reported by Rescorla et al. [14,15] on some dimensional scales. Specifically, we predicted underreporting of problems on both the ASR and ABCL in our Chinese sample due to cultural perceptions of mental illness in China. Such underreporting has been previously documented with respect to mental health in both children and adults [16,17]. Practical and theoretical implications of our findings are also discussed.

## 2. Materials and Methods

### 2.1. Participants and Procedure

The participants in the current study were the parents of children from a larger population-based Jintan Cohort Project, which was established in 2004 in Jintan, Jiangsu province, located in the southeastern region of Mainland China [18,19,20]. The children of the Jintan Cohort project were selected from four pre-schools chosen to be representative of the geographic (rural, urban, and suburban areas), social, and economic profile of Jintan. All participants were Chinese with 99.8% of the cohort being of Han ethnicity. Detailed sample characteristics of this cohort of children were documented in our previous two cohort profile papers [18,19] as well as a methodology paper [20]. We asked the parents of this cohort of children to participate in this instrument validation study. Participants were asked to assess themselves (ASR) (N = 1276) and their spouses (ABCL) (N = 1141) with the Chinese version of the ASR and ABCL when they accompanied their children to take tests for the Jintan project. Some participants completed the ASR/ABCL questionnaires on site, while others completed the ASR/ABCL questionnaires at home and returned them to our laboratory two weeks later. Written informed consent was obtained from parents of the children. Institutional Review Board approval was obtained from the University of Pennsylvania and the Ethics Committee for Research at Jintan Hospital in China.

### 2.2. Measures

The Adult Self-Report (ASR) and Adult Behavior Checklist (ABCL) serve two informants to assess adult behavior. The Adult Self-Report is designed to ask participants to provide information about him/herself. The parallel ABCL is designed to ask collateral informants (e.g., spouse/partner, parents, and friends/roommates) to answer similar questions to describe the ASR target. More specifically, in the current study, spouses of the ASR subject were chosen as informants to fill in the ABCL questionnaire to further assess the individual who completed the ASR.

The ASR contains 120 problem items written at a fifth-grade reading level that respondents rate as 0 = *not true*, 1 = *somewhat or sometimes true*, and 2 = *very true or often true* based on the preceding 6 months. The ABCL obtains ratings from collaterals for 118 problem items, 115 of which are also on the ASR. As detailed by Achenbach and Rescorla [21], ABCL and ASR item ratings are summed to yield scores on eight statistically derived narrow-band syndromes (e.g., Aggressive Behavior), three broad-band scales (e.g., Internalizing, Externalizing, and Total Problems), and six DSM-oriented scales, as well as several other scales not analyzed in this study. Interspersed among the problem items, there are items comprising a Personal Strengths scale. In addition, the ASR and ABCL have a Friends scale and a Spouse/Partner scale, which consist of items not included in the problem items. The ASR and ABCL forms also provided sociodemographic information for the parents of the Jintan Cohort Study regarding gender, age, residence (coded as *rural, urban*, and *suburban*), and education (coded as *Middle School and below, High School graduate or equivalent, Some college, Bachelor’s degree and above*).

U.S. normative data for the ASR and the ABCL were collected in a national household survey [8], with ABCLs completed for 1636 (81%) of the 2020 ASR cases. Alphas were 0.89 to 0.97 for the ASR and ABCL broad-spectrum scales and 0.51–0.88/0.70–0.91 for the ASR/ABCL syndromes. ASR and ABCL items and scales significantly discriminated between demographically similar clinically referred and non-referred samples of adults. U.S. cross-informant ASR–ABCL correlations averaged 0.40 for the empirically based problem scale.

#### Instrument Translation

Achenbach and colleagues, who were the original developers of the ASR and ABCL [8], authorized the first author of this study to translate the instruments into Chinese. The first author of this report, whose research focuses on psychopathology and behavior, is fluent in English and Chinese and led a team of three in the translating process. We followed a multi-stage translation procedure as detailed in our previous papers reporting results from other instruments we translated [22,23,24]. The original ASR and ABCL were first forward-translated from English to Chinese after which a monolingual reviewer examined the content of the Chinese version for accuracy and ambiguity. The Chinese version was then back-translated into English and the resulting English version was compared to the original. Discrepancies between the two English versions were analyzed to determine whether they were a result of the forward or back-translation. If necessary, the translation procedures were adjusted and repeated to correct any errors. The data collected using this Chinese translation of the ASR and ABCL also contributed to the previous multi-society studies referenced earlier [12,13,14,15].

### 2.3. Data Analysis

Following Ivanova et al. [12,13], we conducted CFAs on 99 ASR items and 93 ABCL items. Most of the items were the same on the two CFAs, but some items were tested for the ASR but not the ABCL (and vice versa). Ratings of 2 (*Very True or Often True*) and 1 (*Somewhat or Sometimes True*) were recoded as 1 = present in order to obtain a dichotomous scale. We used the WLSMV estimator, with the root mean square error of approximation (RMSEA), the comparative fit index (CFI), and the Tucker–Lewis index (TLI) as our measures of model fit. Good fit was set at RMSEA ≤ 0.06, CFI ≥ 0.95, TLI ≥ 0.95, based on Hooper et al. [25]. Similarly, excellent fit was set at RMSEA ≤ 0.03 [25]. Next, for the 838 participants with both an ASR and an ABCL, we conducted 2 (informant) × 2 (gender) × 4 (education) mixed-model ANOVAs on all ASR and ABCL Problem scales, as well as on the Personal Strengths, Friends, and Spouse/Partner scales. Effect sizes (ESs) for ANOVAs were measured by η^2^ and characterized using Cohen’s [26] criteria (small = 0.01 to 0.059, medium = 0.06 to 0.139, large > 0.14). Then, we computed cross-informant correlations between the ASR and the ABCL for all scales. We set alpha at *p* < 0.001 and report ESs rather than F and *p* values. Finally, we used independent sample t-tests to compare mean ASR syndrome scores for our Chinese sample with ASR scores from the 17 societies in the Rescorla et al. [14] international sample. We performed similar comparisons for ABCL scores for our Chinese sample and scores for the Rescorla et al. [15] 14-societies sample.

## 3. Results

### 3.1. Demographic Characteristics

Demographic characteristics of the ASR and ABCL samples appear in Table 1. Of the 1276 participants who completed the ASR, four were excluded due to anomalous ages and 256 were excluded because they had >8 missing ASR items, leaving 1016 cases (age range 30 to 56). Of 1142 ABCLs collected, three were excluded for anomalous ages and 231 were excluded because >8 items were missing, leaving 911 forms collected for ASR target adults (age range 30 to 56). The mean ages for both the ASR and the ABCL samples were very similar (ASR: M = 38.02, SD = 0.093; ABCL: M = 38.12, SD = 0.099), which is to be expected as they refer to the same individuals (i.e., ABCL responses refer to ASR targets). We did not subdivide the sample by age for our analyses because respondents fell into a narrow age range, due to the fact that they were all parents of teenagers. About half the ASR respondents were male (47.64%), as were the ASR targets rated by ABCL respondents (50.60%). All couples consisted of a man and a woman. The distribution of education levels was as follows: middle school and below (ASR: 30.76%, ABCL: 32.99%), high school graduate (ASR: 33.91%, ABCL: 33.10%), some college (ASR: 20.61%, ABCL: 19.84%), and college graduate and above (ASR: 14.42%, ABCL: 13.73%). The fact that ABCL percentages were so similar to ASR percentages indicates that the 911 ASR targets for whom ABCLs were obtained were very representative of the full ASR sample of 1016.

### 3.2. CFA Results

#### 3.2.1. Model Fit

Our ASR CFA yielded RMSEA = 0.016, CFI = 0.989, and TLI = 0.988, all indicating excellent model fit. For collateral-reports, we found that RMSEA = 0.018, CFI = 0.996 and TLI = 0.996, also indicating excellent model fit. Therefore, we found that the Jintan data showed excellent fit to the 8-syndrome model for both the ASR and the ABCL.

#### 3.2.2. Factor Loadings

Syndrome loadings for both the self-report and collateral-report showed satisfactory results (Table 2), but ABCL loadings were generally higher than ASR loadings. Mean factor loadings for the ASR syndromes ranged from 0.35 (Thought Problems) to 0.54 (Anxious/Depressed). Nine problem items had loadings below 0.3 (indicating non-significant) on their designated syndrome, five of which were for Rule-Breaking Behavior, three for Aggressive Behavior, and one was for Thought Problems. These non-significant loadings constituted 9.1% of item loadings for the ASR. Mean ABCL loadings of syndromes ranged from 0.64 (Intrusive) to 0.80 (Thought Problems). Most ABCL loadings were within the 0.6–0.8 range. The only non-significant loading (1.1% of all ABCL loadings) was on the Rule-Breaking Behavior syndrome.

### 3.3. Effects of Informant, Gender, and Education on ASR and ABCL Scores

As shown in Table 3, informant effects for nine of the 17 problem scale ESs (η^2^) were small (<6%), six were medium (6–13.9%), and two were large (≥14%). There were no significant effects for gender or education. Of the 51 interactions, 37 were not significant, while the remaining 14 were significant at *p* < 0.001.

A notable finding in Table 3 is that the informant effect was much larger for Internalizing than for Externalizing (16% vs. 3%). Adults self-reported more Internalizing problems on the ASR than their ABCL collaterals reported about them: 7.47 (8.52) vs. 4.10 (6.22). However, ASR and ABCL scores for Externalizing were much more similar: 5.67 (5.73) versus 4.53 (6.51).

This same pattern was found for the syndromes comprising the Internalizing and Externalizing composites. Informant ESs ranged from 8% to 12% for the three Internalizing syndromes (Anxious/Depressed, Withdrawn/Depressed, Somatic Complaints) but only from 1% to 4% for the three Externalizing syndromes (Aggressive Behavior, Rule-Breaking Behavior, and Intrusive). Interestingly, the *DSM*-oriented scales do not show this pattern, as informant ESs only ranged from 2% to 7% across the six scales.

**Figure 1:**
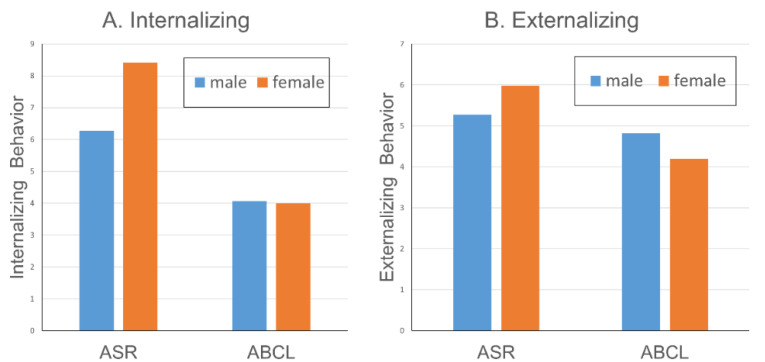
Informant × Gender Interaction on ASR/ABCL Broad-Band Scales.

Although the main effects for gender were not significant, there were small (ESs = 1–2%) but significant informant × gender interactions for 14 of the 17 problem scales, as seen in Table 3. As shown in Figure 1, the ASR showed a larger gender difference for Internalizing than did the ABCL. Collateral-reports yielded comparable scores for men and women targets on Internalizing, but self-reports yielded higher scores for women than men. For Externalizing, the informant × gender interaction depicted in Figure 1 shows a small cross-over effect, such that women self-reported more Externalizing behavior than men, but collaterals reported more Externalizing behavior in men than in women. Many of the syndromes comprising Internalizing and Externalizing showed this same informant × gender interaction pattern.

There was a very large informant effect for the Personal Strength scale (ES = 19%), with ASR scores higher than ABCL scores. Education was also significant (ES = 3%), as higher Personal Strengths scores were associated with more education while the main effects for education were not significant in all other scales. Gender was not significant, but there was a significant informant x gender interaction (ES = 1%), such that the ASR > ABCL differences were smaller for men than for women, with husbands rating their wives’ Personal Strengths the lowest of the four mean scores. No effects were significant for the Friends scale. On the Spouse/Partner scale, the only significant effect was education level, with higher (more favorable) Spouse/Partner scores in dyads where the target adult had more education.

### 3.4. Cross-Informant Agreement Correlations

Our results indicated significant (*p* < 0.01) medium-to-large cross-informant correlations between ASR and ABCL syndrome scales (from 0.41 for Intrusive Behavior to 0.60 for Rule-Breaking Behavior, mean = 0.50), *DSM*-Oriented scales (from 0.45 for Avoidant Personality Problems to 0.60 for Antisocial Personality Problems, mean = 0.51), the Personal Strength scale (0.58), the Friends scale (0.55), and the Spouse/Partner scale (0.46).

### 3.5. Comparison of ASR Scores between the Chinese and International Samples

As shown in Table 4, Chinese mean ASR scores were significantly lower than multicultural mean ASR scores reported by Rescorla et al. [14] for 17 international samples on all problem scales tested. All but four of the *d* values for these comparisons were >0.80, Cohen’s [26] benchmark for large effects. Table 4 also shows that Chinese SDs were much larger than the SDs reported for the international samples. As the latter values are SDs of the multicultural means (obtained by averaging the 17 societal means), they are much smaller than the Chinese SDs, which reflect within-society variation. The Chinese mean for the ASR Spouse/Partner scale was much higher than the multicultural mean for this scale from Rescorla et al. [14], suggesting that the Chinese adults reported better relations with their spouses than did the adults in the 17-society sample. No differences were found for the Personal Strength and Friends scales.

As shown in Table 5, Chinese ABCL problem scores were significantly lower than the multicultural means for the 14-society international sample, with large *d* values. As with the ASR, Chinese SDs were larger than the SDs of the multicultural means in the 14-society international sample, reflecting the commonly found pattern of larger within-society than between-society variation in ASR and ABCL scores. As found for the ASR, the Chinese ABCL Spouse/Partner scale mean score was significantly higher than the multicultural mean for the 14-society sample. Unlike on the ASR, the Chinese means for Friends and Personal Strengths were also significantly higher than the multicultural means for the international sample.

## 4. Discussion

There are currently few standardized, low-cost instruments available for assessing broad psychopathology including emotion and behavior traits in adults. Of those available, few have parallel self-report and collateral-report forms. Furthermore, very few of these measures have been examined in the Chinese population. The results from the current study regarding the syndrome structure, effects of gender, age, and informant on scale scores, and cross-informant agreement for our Chinese translation of the ASR and ABCL contribute to the literature by addressing these gaps. Our findings help to establish that the Chinese ASR/ABCL is a culturally sensitive and valid tool for measuring adult psychopathology.

Our analyses revealed several important findings. Consistent with Ivanova et al. [12,13], we found strong evidence supporting the factor structure of the ASR and ABCL in our adult sample from the Jintan Cohort Study. We found excellent model fit for both ASR self-reports and ABCL collateral-reports. Our ANOVAs yielded no significant effects for either gender or education, indicating that results are comparable across genders and SES/education groups. Higher Personal Strengths scores were also linked to greater levels of education, though the main effects for education were not significant in all other scales. While prior meta analyses have shown small-to-medium mean correlations, our findings revealed medium-to-large cross-informant correlations between the ASR and ABCL syndrome scales [27]. These findings suggest that spouses/romantic partners have high levels of agreement, as prior studies cited in meta analyses have typically utilized a variety of informants (e.g., a mixture of romantic partners, parents, children, and friends/roommates) [27]. However, even when compared to Deluca et al.’s [28] analysis of ASR/ABCL behavior problem scale invariance among young adult romantic couples, our cross-informant correlations remain unusually large. We suspect this may be due to the greater mean age of participants in our study in comparison to that of Deluca et al.’s [28]. Indeed, prior research has suggested that longer relationships may be associated with higher levels of inter-informant agreement, and our findings provide further evidence to support this claim [29].

The ASR yielded more self-reported internalizing conditions than the ABCL, while self-reports of externalizing conditions were similar on the two measures. That scores of Externalizing but not Internalizing were highly similar on the ASR and ABCL could reflect collaterals’ lack of awareness of their spouses’ experiences of internal distress and depression. As is commonly discussed in the literature on psychopathology assessment in children, externalizing behaviors typically are more observable than internalizing behaviors and thus would be expected to yield greater informant agreement [30]. This would explain the much larger informant effect for Internalizing than for Externalizing. However, this is rather inconsistent with Achenbach et al.’s [27] meta-analysis which reported that the mean correlation for externalizing problems did not differ significantly from the mean correlation for internalizing problems. It is possible that internalizing behaviors were more likely to be concealed from others in the Chinese sample due to cultural values that emphasize emotional control [31], a potentially significant contributing factor to the larger informant effect for Internalizing. While the empirically derived scales showed that adults self-reported more internalizing problems than informant reports, the *DSM*-oriented scales did not show this same pattern. We believe there are a number of factors that could be contributing to this difference. First, *DSM*-oriented scales assess narrower constructs than empirically derived scales (i.e., *DSM*-Anxiety and *DSM*-Depression vs. Anxious/Depressed) and are also shorter, so an argument could be made that *DSM*-oriented scales inherently capture less variance. Second, *DSM*-oriented scales appear to assess more observable problems than empirically derived scales (i.e., *DSM*-Depression vs. Anxious/Depressed), which could have driven cross informant agreement up and lowered the informant effect for *DSM*-oriented scales. Finally, this difference could also be due to sampling fluctuations, i.e., unique features of our sample.

Although the main effects for gender were not significant, the significant informant × gender interactions on the majority of problem scales suggest that women in the sample were less likely to disclose their depression, anxiety, and somatic problems to their husbands than vice versa. Perhaps the cultural practice of emotional restraint is exhibited to a greater degree in Chinese women than men; however, it is also possible that there was general underreporting by women in the current sample. Further, the sizable informant effect for the Personal Strengths scale indicates that adults rated their own Personal Strengths higher than did their collaterals. These significant findings seem somewhat inconsistent with the high Spouse/Partner scale scores found on both the ASR and the ABCL for the Chinese sample.

Our comparisons between the Chinese sample and 14-society (multicultural) sample revealed that the Chinese sample reported overall lower scores on the ASR-broad-band, ASR-syndromes, and ASR *DSM*-oriented scales. This underreporting of mental illness by Chinese populations is consistent with previous findings, which demonstrate that the parent-reported prevalence of childhood behavior problems and the adult self-reported prevalence of behavior problems in Chinese samples are lower than those of many other countries [16,17,32]. As alluded to earlier, one possible explanation for the occurrence of underreporting is the stigmatization of mental illness in Chinese culture, in which individuals with mental illness are perceived to be shameful, violent, and dangerous [33]. Another potential explanation for lower mean scores in Chinese samples is the cultural forces that shape the perception of normal versus abnormal behavior across the lifespan. For example, higher levels of internalizing behavior may not necessarily indicate the presence of illness and are rather viewed as a “normal” reaction due to the practice of self-control and emotional restraint emphasized in Chinese societies.

The adults in the Chinese sample appeared to “underreport” most kinds of problems on the ASR and ABCL, but they may have “overreported” on the Spouse/Partner scale, where higher scores are more favorable. Their mean scores were more than double those of the multicultural samples. It is possible that the traditional Chinese emphasis on the resiliency and harmony of the family unit [34] led our Chinese participants to report their spouse/partner relations very favorably. Another possibility is that, given the emphasis placed on the strength of the family in Chinese populations, participants may have provided responses that are socially desirable, therefore contributing to higher scores. On the other hand, the sizable informant effect for the Personal Strengths scale indicates that adults rated their own Personal Strengths higher than did their collaterals, suggesting a somewhat different picture of spousal relations in the Chinese sample than is suggested by the Spouse/Partner scale findings. Additional research is therefore needed to determine the myriad of complex factors that contribute to cultural differences we found between our Chinese ASR and ABCL scores and those reported by Rescorla et al. [14,15] on this scale.

The Rule-Breaking Behavior scale yielded five items with loadings less than 0.3 on the ASR. The concept of “saving face,” prominent in Chinese culture, may have contributed to the issues with this scale. In collectivist cultures, individuals tend to attribute losing face to internal attributes versus individualist cultures in which factors external to one’s ability are stressed [35]. Perhaps individuals were embarrassed to report rule-breaking behaviors, such as “steals,” “uses drugs,” or “trouble with the law” for fear of face-loss. As such, it is possible that the scale was not able to accurately account for this cultural phenomenon, resulting in loading issues with a handful of the problem items.

Our findings support the utility of the ASR and ABCL in Chinese populations and are generally consistent with previous research validating their utility in multinational societies [12,13,14,15]. Results suggest these instruments have an internationally validated factor structure, good cross-informant agreement, and gender patterns consistent with previous research, while also being efficient, economic, and having a low burden in terms of administration. These instruments offer an opportunity to increase research in cross-cultural psychopathology. It is plausible that Western versus non-Western cultural differences in the willingness to self-disclose, collectivism versus individualism, and interpersonal style may affect the manifestation of psychopathology as assessed by standardized rating forms. More research is needed examining the performance of these instruments in adults in Western and non-Western populations in order to better understand culture-specific factors influencing the expression of maladaptive traits and behaviors.

### Limitations and Future Directions

Findings should be interpreted in light of this study’s limitations. Notably, the taxonomy of problem behaviors studied in the current sample is not comprehensive and cannot be generalized to all forms of adult psychopathology in Chinese populations. It is possible that there are behaviors and traits specific to Chinese culture not reflected in these measures, which were originally developed based on data from Western samples. Similarly, Jintan is located in the Southeastern part of China and cannot be fully representative of the broader Chinese population who practice subcultures. Furthermore, our Jintan sample had a narrow age range because all the adults were parents of teenage children, unlike the Rescorla et al. [14,15] international samples, which ranged from 18 to 59 years. To amend this, future testing will need to be conducted with the ASR and ACBL in other populations in additional geographic regions of China and with a wider age range. Several demographic variables were not included in this analysis, including whether the spouses/partners who served as the informants for the ABCL were living with the ASR target at the time the questionnaires were completed. Finally, all informants in the current sample were spouses of the target. Future research could diversify the type of informants used, i.e., utilize a variety of informants as opposed to solely spouses and compare the extent of cross-informant agreement.

## 5. Conclusions

In conclusion, although ASEBA’s ASR and ABCL English versions are widely used, we are the first to report findings of the Chinese version of the ASR and ABCL. This analysis represents an important contribution to the cross-cultural understanding of mental health assessment as it offers a psychometrically sound approach to measuring adult psychopathology in Chinese populations. More research should examine the ASR and ACBL in wider samples of Western and non-Western individuals to further improve the generalizability of the findings reported here.

Furthermore, we would also like to emphasize the importance of the clinical implications of these instruments outside of research, as they may serve as initial routine screening tools in various health settings to improve quality of care for adults in multiple dimensions of health outcomes, including behavioral, social, emotional, and thought problems. Particularly, in the area of early prevention, they provide clinicians with a useful, yet cost-effective, baseline assessment off of which to make decisions regarding type and level of care.

## Figures and Tables

**Table 1:** Sample Characteristics of Self-Report (ASR) and Collateral-Report (ABCL).

Characteristics	ASR (N = 1016)		ABCL (N = 911)	
Age	38.023 ± 0.093		(mean) 38.123 ± 0.099	
Gender				
Father	47.64%		50.60%	
Mother	52.36%		49.40%	
Location				
Rural	169	16.63%	155	17.01%
Urban	413	40.65%	370	40.61%
Suburban	434	42.72%	386	42.37%
Total	1016		911	
Education				
Middle School and below	303	30.76%	286	32.99%
High School graduate or equivalent	334	33.91%	287	33.10%
Some college;	203	20.61%	172	19.84%
Bachelor’s degree and above	142	14.42%	119	13.73%
Other	3	0.30%	3	0.35%
Total	985		867	

**Table 2:** Descriptive Statistics for Factor Loadings of ASR and ABCL.

ASR/ABCL Syndromes	ASR Factor Loading	ABCL Factor Loading
***Anxious/Depressed***	***0.54***	***0.78***
12. Lonely	0.56	0.77
13. Confused	0.61	
14. Cries a lot	0.40	0.79
22. Worries about future	0.54	
31. Fears doing bad	0.41	0.66
33. Feels unloved	0.56	0.77
34. Others out to get them	0.49	0.77
35. Feels worthless	0.59	0.81
45. Nervous, tense	0.58	0.79
47. Lacks self-confidence	0.61	0.78
50. Fearful, anxious	0.53	0.87
52. Feels too guilty	0.57	0.77
71. Self-conscious	0.56	0.68
91. Suicidal thoughts	0.34	
103. Unhappy, sad	0.64	0.84
107. Cannot succeed	0.63	0.79
112. Worries	0.62	0.78
113. Worries about my relations with the opposite sex	0.49	
***Withdrawn***	***0.52***	***0.77***
25. Does not get along	0.58	0.84
30. Poor relations with opposite sex	0.47	0.79
42. Rather be alone	0.47	0.62
48. Not liked	0.52	0.75
60. Enjoys little	0.50	0.80
65. Refuses to talk	0.55	0.86
67. Trouble making friends	0.54	0.87
69. Secretive	0.54	0.65
111. Withdrawn	0.54	0.78
***Somatic Complaints***	***0.49***	***0.75***
51. Feels dizzy	0.61	0.85
54. Tired without reason	0.66	0.86
56a. Aches, pains	0.52	0.86
56b. Headaches	0.50	0.66
56c. Nausea, feels sick	0.47	0.90
56d. Eye problems	0.39	0.63
56e. Skin problems	0.33	0.66
56f. Stomach aches	0.39	0.58
56g. Vomiting	0.43	0.78
56h. Heart pounding	0.51	
56i. Numbness	0.54	
100. Trouble sleeping	0.46	
***Thought Problems***	***0.35***	***0.80***
9. Cannot get mind off thoughts	0.46	0.68
18. Harms self	0.30	0.87
36. Accident-prone	0.42	
40. Hears sounds, voices	0.25 ^#^	0.71
46. Twitching	0.34	
63. Prefers older people	0.36	
66. Repeats acts	0.39	0.81
70. Sees things	0.31	0.86
80. Stares blankly ^		0.61
84. Strange behavior	0.35	0.94
85. Strange ideas	0.32	0.88
91. Suicidal thoughts ^		0.88
***Attention Problems***	***0.49***	***0.71***
1. Forgetful	0.43	0.57
8. Cannot concentrate	0.54	0.69
11. Too dependent	0.51	0.61
13. Confused ^		0.84
17. Daydreams	0.43	0.74
53. Trouble planning	0.59	0.78
59. Fails to finish	0.54	0.80
61. Poor work performance	0.44	0.82
64. Trouble setting priorities	0.49	0.80
78. Trouble making decisions	0.63	0.74
96. Lacks initiative ^		0.75
101. Skips job	0.48	0.64
102. Lacks energy	0.60	0.80
105. Disorganized	0.44	0.72
108. Loses things	0.44	0.58
119. Not good at details	0.48	0.65
121. Late for appointments	0.35	0.63
***Aggressive Behavior***	***0.45***	***0.76***
3. Argues	0.36	0.60
5. Blames others	0.38	0.68
16. Mean to others	0.36	0.73
28. Gets along badly with family	0.43	0.80
37. Gets in fights	0.23 ^#^	0.85
55. Mood swings between elation and depression	0.63	0.84
57. Attacks people	0.28 ^#^	0.81
68. Screams a lot	0.33	0.72
81. Changeable behavior	0.46	0.68
86. Stubborn, sullen, irritable	0.64	0.82
87. Mood changes	0.64	0.83
95. Hot temper	0.54	0.69
97. Threatens people	0.27 ^#^	0.80
113. Sulks ^		0.80
116. Easily upset	0.67	0.78
118. Impatient	0.57	0.73
***Rule-Breaking Behavior***	***0.35***	***0.68***
6. Uses drugs	0.28 ^#^	0.51
20. Damages own things	0.38	
23. Breaks rules	0.39	0.81
26. Lacks guilt	0.40	0.77
39. Bad friends	0.29 ^#^	0.80
41. Impulsive	0.55	0.72
43. Lying, cheating	0.38	0.78
76. Irresponsible	0.34	0.87
82. Steals	0.18 ^#^	0.20 ^#^
90. Gets drunk	0.23 ^#^	0.51
92. Trouble with the law	0.22 ^#^	0.70
114. Fails to pay debts	0.42	0.83
117. Trouble managing money	0.51	0.69
122. Trouble keeping jobs	0.41	0.69
***Intrusive***	***0.44***	***0.64***
7. Brags	0.31	0.66
19. Demands attention	0.45	0.47
74. Showing off, clowning	0.48	0.71
93. Talks too much	0.51	0.55
94. Teases a lot	0.38	0.74
104. Loud	0.50	0.74

Notes: 1. CFAs were conducted on 99 ASR items and 93 ABCL items. Most of the items were the same on the two CFAs, but some items were tested for the ASR but not the ABCL (and vice versa), as indicated by empty cells. Values in bold and italics refer to mean factor loadings for the ASR and ABCL syndromes. 2. ^ indicates the five items which are included in ASR but not in ABCL 3. ^#^ indicates the non-significant item loading (loadings below 0.3) in the ASR/ABCL.

**Table 3:** Effect Sizes (η^2^) for Informant (I), Education (E), and Gender (G) on the ASR and ABCL.

Scale	ASR–ABCL Cross Informant Results						
	I	E	G	I × E	I × G	G × E	I × G × E
Broad-Band Scales							
Total Problems	11%	ns	ns	ns	1%	ns	ns
Internalizing	16%	ns	ns	ns	2%	ns	ns
Externalizing	3%	ns	ns	ns	1%	ns	ns
Syndromes							
Anxious/Depressed	12%	ns	ns	ns	2%	ns	ns
Withdrawn/Depressed	8%	ns	ns	ns	ns	ns	ns
Somatic Complaints	12%	ns	ns	ns	1%	ns	1%
Thought Problems	18%	ns	ns	ns	ns	ns	ns
Attention Problems	1%	ns	ns	ns	2%	ns	ns
Aggressive Behavior	2%	ns	ns	ns	2%	ns	ns
Rule-Breaking Behavior	1%	ns	ns	ns	2%	ns	ns
Intrusive Behavior	4%	ns	ns	ns	ns	ns	ns
*DSM*-Oriented Scales							
Depressive Problems	2%	ns	ns	ns	3%	ns	ns
Anxiety Problems	4%	ns	ns	ns	1%	ns	ns
Somatic Problems	6%	ns	ns	ns	2%	ns	ns
Avoidant Personality Problems	7%	ns	ns	ns	2%	ns	ns
Attention Deficit Hyperactivity Problems	2%	ns	ns	ns	2%	ns	ns
Antisocial Personality Problems	2%	ns	ns	ns	1%	ns	ns
Other Scales							
Personal Strengths	19%	3%	ns	ns	ns	ns	ns
Friends	ns	ns	ns	ns	ns	ns	ns
Spouse/Partner	ns	ns	ns	ns	ns	ns	ns

Note: ns indicates the effect was not significant; Interactions (not shown in table) were not significant or had an effect size of <1%; I = informant, E = education, G = gender.

**Table 4:** Mean Score Comparisons and Effect Sizes for Chinese and 17 Societies on the ASR.

	China (N = 1016)		17 Societies (N = 10,197)			
ASR Scale	Mean	SD	Mean	SD	t	d′
Broad-Band Scales						
Total Problems	21.3	20.6	42.7	6.1	76.52 **	1.41
Internalizing	6.9	8.0	14.6	2.1	74.75 **	1.32
Externalizing	5.6	5.8	10.5	1.8	60.85 **	1.14
Syndromes						
Anxious/Depressed	3.5	4.4	8.0	1.1	80.98 **	1.40
Withdrawn	1.5	2.1	3.3	0.8	55.23 **	1.13
Somatic Complaints	1.9	2.6	3.3	0.7	41.38 **	0.73
Attention Problems	3.5	3.7	6.6	0.7	72.59 **	1.16
Aggressive Behavior	3.0	3.2	5.3	1.0	51.59 **	0.97
Intrusive Behavior	1.2	1.5	2.4	0.6	50.05 **	1.05
*DSM*-Oriented Scales						
Depressive Problems	2.4	3.1	4.9	0.6	69.44 **	1.12
Somatic Problems	1.3	2.0	2.2	0.6	32.94 **	0.61
Avoidant Personality Problems	1.6	1.9	3.0	0.4	61.92 **	1.02
Attention Deficit Hyperactivity Problems	2.9	3.1	5.7	0.7	74.20 **	1.25
Antisocial Personality Problems	2.5	3.0	4.2	1.0	39.35 **	0.76
Other Scales						
Personal Strengths	14.2	5.0	15.5	2.1	15.78 *	0.34
Friends	7.8	2.8	8.2	0.7	11.31 *	0.20
Spouse/Partners	8.6	3.3	4.3	0.8	−104.380 **	−1.79

** *p* < 0.001; * *p* < 0.05.

**Table 5:** Mean Score Comparisons and Effect Sizes for Chinese and 14 Societies on the ABCL.

	China (N = 911)		14 Societies (N = 8322)			
ABCL Scale	Mean	SD	Mean	SD	t	d′
Broad-Band Scales						
Total Problems	16.23	20.85	34.4	8.2	51.19 **	1.15
Internalizing	4.18	6.51	9.4	2.2	51.18 **	1.07
Externalizing	4.52	6.38	9.3	2.7	42.10 **	0.98
Syndromes						
Anxious/Depressed	2.04	3.31	4.9	1.2	53.14 **	1.15
Withdrawn	1.14	2.05	2.6	0.8	42.02 **	0.94
Somatic Complaints	1.00	1.88	1.9	0.3	39.35 **	0.67
Thought Problems	0.38	1.14	.8	0.2	29.70 **	0.51
Attention Problems	3.05	4.03	5.9	1.1	49.78 **	0.96
Aggressive Behavior	2.45	3.55	4.8	1.3	40.49 **	0.88
Rule-Breaking Behavior	1.18	2.26	2.4	0.9	31.48 **	0.71
Intrusive Behavior	0.89	1.29	2.1	0.6	49.60 **	1.20
*DSM*-Oriented Scales						
Depressive Problems	2.10	3.21	4.1	0.8	45.41 **	0.85
Anxiety Problems	1.08	1.61	4.1	1.2	69.43 **	2.13
Somatic Problems	0.80	1.60	1.3	0.2	26.68 **	0.44
Avoidant Personality Problems	1.08	1.78	2.3	0.5	47.68 **	0.93
Attention Deficit Hyperactivity Problems	2.29	3.08	4.7	1.1	48.52 **	1.04
Antisocial Personality Problems	1.99	3.32	3.8	1.2	33.59 **	0.73
Other Scales						
Personal Strengths	12.07	5.57	15.3	2.0	35.85 **	0.77
Friends	7.63	3.06	8.0	0.8	8.66 **	0.17
Spouse/Partners	8.59	3.37	4.0	0.8	100.98 **	−1.87

** *p* < 0.001.

## Data Availability

The data presented in this study are available on request from the corresponding author.
